# A Lightweight and Explainable AI Framework Toward Automated Infraocclusion Detection in Pediatric Panoramic Radiographs

**DOI:** 10.3390/diagnostics16060866

**Published:** 2026-03-14

**Authors:** Zeliha Hatipoglu Palaz, Ecem Elif Cege, Bamoye Maiga, Yaser Dalveren, Gonca Gokce Menekse Dalveren, Ali Kara, Ahmet Soylu, Mohammad Derawi

**Affiliations:** 1 Department of Pediatric Dentistry, Faculty of Dentistry, Gazi University, 06490 Ankara, Turkey; zelihahatipoglu@gazi.edu.tr; 2 Department of Pediatric Dentistry, Faculty of Dentistry, Karabuk University, 78050 Karabuk, Turkey; elifcege@karabuk.edu.tr; 3 RST Technology, Hacettepe University Teknokent, 06800 Ankara, Turkey; bamoyemeiga@gmail.com; 4 Department of Electrical and Electronics Engineering, Izmir Bakircay University, 35665 Izmir, Turkey; yaser.dalveren@bakircay.edu.tr; 5 Department of Computer Engineering, Izmir Bakircay University, 35665 Izmir, Turkey; gonca.dalveren@bakircay.edu.tr; 6 Department of Electrical and Electronics Engineering, Gazi University, 06570 Ankara, Turkey; akara@gazi.edu.tr; 7 School of Doctoral Studies, Kristiania University of Applied Sciences, 0107 Oslo, Norway; ahmet.soylu@kristiania.no; 8 Department of Electronic Systems, Norwegian University of Science and Technology, 2815 Gjovik, Norway

**Keywords:** infraocclusion, artificial intelligence, deep learning, detection, classification, panoramic radiographs

## Abstract

**Background/Objectives**: Infraocclusion in pediatric patients may result in space loss, malocclusion and the need for complex orthodontic treatment if not detected early. Conventional diagnosis may be subject to human error and can be challenging, particularly in pediatric cases. The aim of this study is to design and evaluate a lightweight, two-stage deep learning framework with integrated explainable AI (XAI) techniques for automated infraocclusion detection in pediatric panoramic radiographs. **Methods**: Annotated panoramic radiographs of pediatric patients aged 7–11 years were used for training and validation. In the first stage, a MobileNet V2 Lite model was used to detect the region of interest (ROI) comprising premolars and molars. In the second stage, a custom CNN classifier was proposed to distinguish between infraocclusion and no infraocclusion. Model performance was evaluated in terms of diagnostic accuracy, computational complexity, and statistical significance. XAI techniques were also incorporated to visualize model attention and enhance interpretability. **Results**: The detection stage achieved high reliability with a precision, recall, F1-score, and AP50 values of 0.99, and an AP75 of 0.89, indicating accurate ROI localization. The classification stage reached an overall accuracy of 98.78%, with class-specific accuracies of 99.25% for infraocclusion and 98.31% for no infraocclusion cases. The framework also demonstrated computational efficiency, requiring only 1.88 M trainable parameters (7.19 MB), with short training times and low inference latency (0.8 ms for classification and 19 ms for detection). XAI visualizations consistently highlighted clinically relevant regions, such as occlusal margins and interproximal areas, confirming the model’s alignment with radiographic features recognized by clinicians. **Conclusions**: The proposed two-stage framework provides an accurate, computationally efficient, and interpretable solution for automated infraocclusion detection in pediatric patients. Its modular design and reduced complexity support practical integration into routine clinical workflows, including resource-constrained environments. These findings indicate that lightweight and XAI systems may enhance early infraocclusion detection while maintaining clinical transparency.

## 1. Introduction

### 1.1. Motivation

Infraocclusion, also referred to as tooth ankylosis or submerged deciduous teeth, is a developmental anomaly in which a primary molar fails to maintain its normal occlusal level, resulting in infra-position relative to adjacent teeth [1,2]. This condition is clinically significant, as it may lead to occlusal disturbances, loss of arch space, impaction of permanent successors, and asymmetrical eruption patterns if not diagnosed and managed in a timely manner [3]. Early recognition is therefore critical to prevent long-term complications and to support appropriate orthodontic and restorative treatment planning.

Conventional diagnosis of infraocclusion relies on clinical inspection and radiographic evaluation, particularly panoramic or retroalveolar images [4]. However, interpretation of these images can be challenging due to overlapping anatomical structures and variable image quality, as panoramic radiographs often present distortions, ghost shadows, and anatomical superimpositions that complicate clinical interpretation [5]. Moreover, panoramic imaging lacks the fine detail resolution necessary to consistently detect mild infraocclusion. These limitations may contribute to misdiagnosis or delayed recognition in clinical practice [6].

In recent years, artificial intelligence (AI), particularly deep learning-based computer vision approaches, has demonstrated remarkable potential in panoramic radiology. Applications range from automated tooth detection and numbering [7,8] to the identification of caries [9,10], periodontal bone loss [11], impacted teeth [12,13], and eruption anomalies [14]. Given the rapid technological progress in this domain, the integration of AI into pediatric dentistry, particularly in infraocclusion detection, is also expected to be a significant future trend [15]. In parallel, explainable AI (XAI) methods have emerged as an essential complement to deep learning, allowing clinicians to visualize and validate the decision-making process of models, thereby increasing transparency and clinical trust in AI-driven diagnostics [16]. Nevertheless, successful real-world adoption requires more than high diagnostic accuracy. Practical implementation demands computationally efficient, interpretable, and modular systems capable of operating reliably in resource-constrained environments while seamlessly integrating into existing clinical workflows [17].

### 1.2. Related Works

Convolutional neural networks (CNNs) have demonstrated high diagnostic performance across multiple medical imaging domains, including radiology, dermatology, ophthalmology, and digital pathology [18]. In dentistry, AI-driven image analysis has also been increasingly adopted for diagnostic and screening purposes, including oral lesion assessment and radiographic interpretation [19].

Several recent studies have applied AI methods to pediatric dentistry for the detection of oral conditions and anomalies. Deep learning approaches have been employed to identify dental plaque on primary teeth using CNN frameworks, which are designed to automatically learn hierarchical image features for classification and detection tasks [20]. Automated systems for deciduous tooth detection and numbering in panoramic radiographs have also been proposed, where object detection models such as Faster Region-based CNN (Faster R-CNN) and You Only Look Once (YOLO) architectures are utilized for accurate localization and enumeration [21]. In addition, CNN-based models have been investigated for the early detection of supernumerary teeth in pediatric patients during mixed dentition, highlighting their potential for anomaly screening [22]. Other research has focused on the automated classification of early childhood caries using automated machine learning (AutoML). This framework enables the automatic selection and optimization of machine learning approaches and demonstrates the feasibility of machine learning-based risk prediction from clinical and behavioral data [23]. Beyond disease detection, deep neural networks have been developed for estimating chronological age in pediatric and adolescent populations using dental and skeletal features, offering more precise alternatives to traditional methods [24]. Furthermore, AI models have been designed to assist in identifying ectopic eruption of maxillary molars from panoramic radiographs, addressing a diagnostic task that often depends on clinical experience [25]. These works demonstrate the growing role of AI in pediatric dental diagnostics, with diverse methodologies being explored to improve accuracy, reproducibility, and clinical decision support.

Despite the rapid progress in AI-based pediatric dental diagnostics, the detection of infraocclusion in pediatric panoramic radiographs has received very limited attention from the AI research community. Only a pilot study presented in [26] investigated the use of deep learning for identifying submerged deciduous teeth in panoramic radiographs of pediatric patients aged 5 to 12 years. In that work, a moderately deep CNN trained on a relatively small dataset achieved an accuracy of 83.3% and a specificity of 96.8%, indicating satisfactory discriminatory ability for detecting submerged molars. While pioneering, the study was based on a relatively small dataset and did not assess computational complexity or practical deployment considerations. More recently, two-stage deep learning frameworks, typically combining an object detection backbone with a classification network, have been applied in dental image analysis to improve localization and diagnostic accuracy [27]. These studies demonstrate that modular detection–classification approaches may enhance performance by isolating the region of interest (ROI) before classification. However, such architectures are often computationally intensive and primarily optimized for predictive performance rather than lightweight deployment. Furthermore, explainability mechanisms are not consistently integrated, which may limit clinical interpretability. In the specific context of infraocclusion, there remains a lack of systematically evaluated, computationally efficient, and clinically oriented two-stage frameworks supported by comparative analysis against established architectures.

### 1.3. The Scope and Contributions

This study presents a two-stage deep learning approach designed specifically for infraocclusion detection in pediatric patients aged 7 to 11 years. Rather than proposing a novel CNN architecture, the primary objective is to design and systematically evaluate a lightweight, modular, and clinically oriented detection–classification pipeline optimized for resource-constrained environments. In the first stage, a computationally efficient object detector, namely MobileNet V2 Lite, is employed to automatically localize ROI comprising premolars and molars in panoramic radiographs. In the second stage, cropped regions are classified as infraocclusion or no infraocclusion using a custom CNN. This modular structure allows independent optimization of localization and classification components and facilitates integration with alternative backbones when required. Importantly, in contrast to prior AI-based infraocclusion detection study [26], the proposed framework is evaluated not only in terms of diagnostic accuracy but also with respect to computational complexity, including parameter count, model size, training duration, and inference latency. Furthermore, comparative analyses are conducted against widely used deep architectures, including EfficientNet variants, Inception V3, and ResNeXt50-based models, to contextualize performance in both accuracy and efficiency dimensions. Furthermore, to enhance clinical interpretability, XAI techniques, such as gradient-weighted class activation mapping (Grad-CAM), SmoothGrad, and occlusion sensitivity mapping (OSM), are incorporated to visualize model attention and support radiographic validation by practitioners. The primary aim of this work is therefore to evaluate the feasibility and potential clinical applicability of a lightweight two-stage deep learning framework for early screening and diagnosis of infraocclusion in pediatric patients. By prioritizing balanced diagnostic performance, computational efficiency, and interpretability, this work contributes toward the development of deployable AI-assisted tools for pediatric dental care.

The main contributions of this study can be summarized as follows:

- A computationally efficient two-stage deep learning framework is designed and evaluated for infraocclusion detection in pediatric panoramic radiographs.
- The proposed approach emphasizes lightweight architecture, reduced parameter complexity, and low inference latency while maintaining high diagnostic performance.
- Extensive comparative analyses against established classification and detection backbones are conducted to position the framework within current deep learning approaches.
- XAI techniques are integrated to enhance interpretability and support clinical validation.

## 2. System Architecture

**Figure 1:**
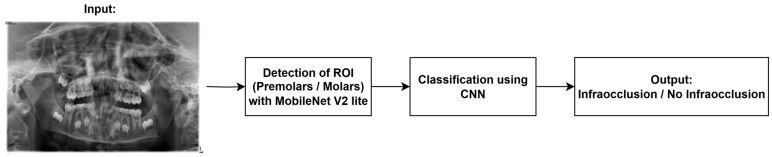
The overall workflow.

The proposed framework employs a two-stage deep learning approach designed to achieve accurate and automated detection of infraocclusion from pediatric panoramic radiographs. The overall workflow is illustrated in Figure 1. Input panoramic radiographs are first processed by the detection module, which localizes the ROI comprising premolars and molars. The extracted ROI is then forwarded to the classification network, which focuses exclusively on the localized teeth and performs binary classification to determine the presence of infraocclusion.

The two-stage design was adopted to enhance diagnostic reliability. By decoupling localization and classification, the framework reduces background interference from surrounding anatomical structures and ensures that the classifier operates solely on standardized tooth-centered image patches. This modular structure may also improve interpretability by allowing independent evaluation of detection and classification performance.

### 2.1. Stage 1: Detection of ROI

The first stage involves the automated detection of the ROI corresponding to the premolars and molars. The original input panoramic radiographs, acquired at a high resolution of 2440 × 1292 pixels, were initially processed for this task. The detection network, which has an integrated preprocessing algorithm in its pipeline, resizes the images to 640 × 640 pixels in order to meet the input constraints while preserving computational efficiency. For the detection task, an object detection framework utilizing a MobileNet V2 Lite backbone was employed. This architecture was selected owing to its optimal balance between computational efficiency and detection accuracy, making it highly suitable for real-time and resource-constrained clinical environments [28]. The detection model identifies the bounding box coordinates of the target teeth. Subsequently, these bounding boxes are used to dynamically crop the high-resolution original images, extracting the precise ROIs that are forwarded to the next classification stage.

### 2.2. Stage 2: Infraocclusion Classification

**Figure 2:**
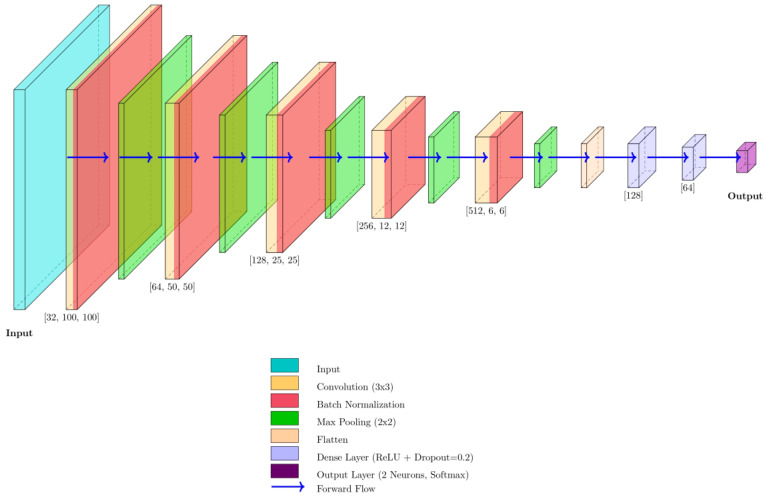
Architecture of the classification network.

Following ROI detection, the cropped image patches were resized to 100 × 100 pixels and processed by a custom CNN designed for binary classification. The classification task was defined as follows: (a) Class 0: Infraocclusion, (b) Class 1: No infraocclusion. The CNN architecture, presented in Figure 2, comprises five convolutional layers with filter sizes progressively increasing from 32 to 512.

Each convolutional layer employs a kernel size of 3 × 3 with stride 1 and standard padding to preserve spatial resolution. Following each convolution, a Rectified Linear Unit (ReLU) activation function introduces non-linearity, enabling the network to model complex anatomical variations. Batch normalization is applied after each convolutional block to stabilize gradient propagation, accelerate convergence, and mitigate internal covariate shift. A max-pooling operation of size 2 × 2 is then applied to progressively down-sample the spatial dimensions of the feature maps (from 100 × 100 down to 6 × 6) while preserving the most salient structural information. This hierarchical design enables the network to learn increasingly abstract representations, ranging from basic edges in the early layers to complex morphological patterns associated with infraocclusion in deeper layers.

Following the convolutional backbone, the resulting feature maps are flattened into a one-dimensional vector to enable transition into the fully connected layers. The classification head includes two dense layers with 128 and 64 neurons, respectively, both activated with ReLU. Between these layers, dropout regularization with a probability of 20% is applied to reduce the risk of overfitting and improve generalization performance. The final output layer consists of two neurons activated by the softmax function, producing probabilistic predictions for the two target classes: infraocclusion and no infraocclusion.

## 3. Materials

### 3.1. Dataset

The study was conducted in accordance with the Declaration of Helsinki and approved by the Ethics Committee of Gazi University, Ankara, Turkey (approval number: 2026-105). Panoramic radiographs of pediatric patients aged between 7 and 11 years, taken between January 2019 and December 2022, were retrospectively collected from the Department of Pediatric Dentistry, Faculty of Dentistry, Gazi University. Radiographic records were accessed through the Picture Archiving and Communication System (PACS) integrated within the institutional Hospital Information Management System. All patients had previously presented for routine dental treatments, and their radiographic records were retrieved for evaluation. Informed consent was obtained from the legal guardians of all participants included in the study. Before the analysis, all radiographic images were anonymized by removing personal identifiers and metadata to ensure patient confidentiality. Data were stored and processed within a secure institutional infrastructure, with access restricted to authorized research personnel only.

A total of 11,246 panoramic radiographs of pediatric patients aged 7–11 years, corresponding to 11,246 unique pediatric patients, were initially screened. After applying the predefined exclusion criteria, 419 radiographs were excluded. The remaining 10,827 radiographs constituted the eligible pool for infraocclusion assessment. Within this screened population, infraocclusion was identified in 450 cases, corresponding to an observed prevalence of 4.15% among the 7–11-year-old age group in the institutional clinical setting. This prevalence falls within the previously reported range of 2.8–41.8% in the literature [29].

Exclusion criteria were defined as follows: (1) patients younger than 7 years or older than 11 years; (2) presence of systemic conditions potentially affecting dental development; (3) primary molars with significant structural loss due to extensive restorations, caries, or wear; (4) unerupted or partially erupted first permanent molars; (5) absence of adjacent teeth that serve as references for identifying infraocclusion; and (6) radiographs with insufficient image quality preventing reliable diagnostic assessment of the entire dental arch.

All panoramic radiographs were acquired using the same radiographic unit (Sirona Orthophos XG 5, Dentsply Sirona, Bensheim, Germany) under standardized exposure conditions of 62 kVp, 8 mA, and 14 s, with a magnification factor of 1.33. Positioning of patients followed the manufacturer’s guidelines to ensure reproducibility. The radiographs were interpreted independently at different times by two experienced examiners under semi-dark conditions using a high-resolution LCD monitor. Disagreements were resolved by consensus to establish the final diagnosis. As the final dataset was determined based on consensus decisions, a formal inter-examiner agreement analysis was not performed.

Cases of infraoccluded primary molars were categorized according to the classification system proposed by Brearley and McKibben [30]. More specifically, infraocclusion was defined according to established criteria in the pediatric dentistry literature, namely when the occlusal surface of a tooth was positioned apically relative to the adjacent occlusal plane by approximately 1 mm or more. Radiographs in which no such discrepancy was present were classified as normal. This diagnostic boundary was applied consistently, and all cases were reviewed by the first two authors to ensure accuracy.

For model development, an image-level dataset was first constructed from the eligible pool for the detection stage. To mitigate class imbalance effects during deep learning training, a balanced subset was created for the detection stage. The detection dataset consisted of 600 panoramic images, with 300 images containing infraocclusion and 300 images with no infraocclusion. This dataset was used to train the object detection network to localize the ROI. The detection dataset was partitioned at the subject level into 70% training and 30% testing subsets. The trained detection model was designed to localize the premolar and molar regions within each panoramic radiograph. As each radiograph yielded two ROIs, the 600 images in the detection dataset generated a total of 1200 ROI-level samples. Among these, 450 ROIs corresponded to infraocclusion cases.

For the classification stage, all 450 infraocclusion ROIs were included. To achieve class balance, 450 no-infraocclusion ROIs were randomly selected from the remaining negative ROI pool, resulting in a classification dataset comprising 900 ROI-level samples. The classification dataset was partitioned at the subject level into 70% training, 15% validation, and 15% testing subsets.

To prevent data leakage and artificial performance inflation, dataset partitioning was performed at the subject level prior to any augmentation procedures. All radiographs belonging to a given patient were assigned exclusively to a single subset (training, validation, or testing). Augmentation was subsequently applied to the ROIs generated by the detection stage. Importantly, all augmented variants derived from a specific subject were retained within the same dataset split to ensure strict separation between subsets.

### 3.2. Data Augmentation

Data augmentation was applied after subject-level dataset partitioning in order to eliminate the risk of data leakage across training, validation, and testing subsets. Augmentation was performed at the ROI extracted by the detection stage. Each cropped image was treated as an independent sample for transformation. However, all augmented variants derived from a specific subject were retained within the same dataset split.

For the detection dataset, augmentation techniques including random horizontal flipping, brightness adjustment, and scaling were employed to increase robustness to geometric and illumination variability, resulting in an effective expansion from 600 to 1200 images for training purposes.

For the classification dataset, augmentation was applied in a controlled and non-exhaustive manner. Instead of generating all possible combinations of transformations, each sample was randomly subjected to two or three augmentation operations selected from a predefined transformation pool. These transformations included image rotation within the range of −20° to +20°, vertical flipping, and contrast variation within a factor range of 0.1 to 0.5. This augmentation strategy was adopted to enhance variability while avoiding the creation of excessive near-duplicate samples that could artificially inflate performance metrics. Class balance was carefully maintained throughout the augmentation process. Identical transformation policies were applied to both infraocclusion and no-infraocclusion classes to maintain a balanced distribution across subsets. The final classification dataset consisted of 2667 samples, as summarized in Table 1.

### 3.3. Training Details

The networks considered in this study were optimized using the Adam optimizer with an initial learning rate of 0.0025. A learning rate scheduler was applied to reduce the learning rate by a factor of 0.1 based on validation loss, with a patience of five epochs. Early stopping was employed with a patience of five epochs to prevent overfitting and improve generalization.

For the detection stage, training was conducted for a maximum of 2000 epochs, while for the classification stage, training was performed for up to 60 epochs. In both stages, early stopping was activated when no improvement in validation loss was observed within the defined patience window. The training process was guided by categorical cross-entropy loss for the classification stage, while detection performance was optimized using the corresponding detection loss function.

### 3.4. Evaluation Metrics

To ensure a rigorous evaluation, multiple performance metrics were employed for both the detection and classification tasks. For the evaluation of detection accuracy, primary performance metrics, specifically Precision, Recall (Sensitivity), and the F1–score, were employed:

(1) $$Precision=\frac{TP}{TP+FP},$$

(2) $$Recall=\frac{TP}{TP+FN},$$

(3) $$F1–score=2\times \frac{Precision\times Recall}{Precision+Recall},$$

where TP, FP, and FN denote true positives, false positives, and false negatives, respectively. Together, these metrics provide a balanced assessment of the model efficiency in correctly identifying infraocclusion cases [31]. Precision reflects the proportion of correctly identified positive detections among all positive predictions, while Recall measures the proportion of correctly identified positive detections among all ground-truth positives. The F1–score provides a harmonic mean, offering a more robust indicator when there is an imbalance between Precision and Recall.

Moreover, to evaluate the detection performance, Average Precision (AP) at different Intersection over Union (IoU) thresholds was used. IoU is defined as the ratio between the area of overlap and the area of union between the ground truth (A) and predicted bounding box (B):

(4) $$IoU=\frac{\left|A\cap B\right|}{\left|A\cup B\right|},$$

where an IoU value of 1.0 indicates perfect overlap, whereas 0 indicates no overlap. In object detection, a prediction is considered a TP only if its IoU with the corresponding ground truth exceeds a predefined threshold. For this reason, AP was calculated at fixed IoU thresholds of 0.50 and 0.75, reported as AP50 and AP75, respectively. Specifically, AP50 corresponds to detection performance under a moderate localization requirement (IoU≥ 0.50), while AP75 applies a stricter overlap criterion (IoU≥ 0.75) that provides a more rigorous assessment of bounding box localization quality [32].

On the other hand, for the classification stage, overall Accuracy, Specificity, Recall expressed in Equation (2), F1–score expressed in Equation (3), and the Area Under the ROC Curve (AUC) were adopted as the primary metric [33]:

(5) $$Accuracy=\frac{TP+TN}{TP+TN+FP+FN},$$

(6) $$Specificity=\frac{TN}{TN+FP},$$

where TN represents true negatives. Here, the AUC is used to assess the overall discriminatory power across all possible classification threshold. It is mathematically defined as the integral of the True Positive Rate (TPR) against the False Positive Rate (FPR):

(7) $$AUC=\int_{0}^{1}TPR(FPR)dFPR$$

where

(8) $$TPR=\frac{TP}{TP+FN},$$

and

(9) $$FPR=\frac{FP}{FP+TN}.$$

Additionally, categorical cross-entropy loss was monitored across training, validation, and testing sets. Accuracy and loss convergence curves were analyzed to provide insight into both the stability of training and the generalization capacity of the model. Together, these metrics allowed for a comprehensive assessment of model robustness and diagnostic reliability.

In addition to accuracy-based measures, computational performance was also evaluated to capture the efficiency of the proposed framework. The assessment included training time, number of trainable parameters, model size, and inference time, which are widely adopted indicators for quantifying model complexity and feasibility in real-world applications. Training time reflects the efficiency of the learning process, while the number of parameters and model size characterize the memory footprint and scalability of the architecture [34]. Inference time was measured as the average latency required to process a single image, providing an estimate of the framework’s suitability for real-time deployment in clinical workflows [35].

### 3.5. Implementation

All experiments were conducted on a workstation equipped with an NVIDIA GeForce RTX 3070 GPU with 16 GB of dedicated memory, a 3.6 GHz 12-core CPU, and 32 GB of RAM, operating on Windows 11. The models were implemented in Python 3.9, leveraging TensorFlow for model development, training, and evaluation.

## 4. Results

### 4.1. Model Convergence

**Figure 3:**
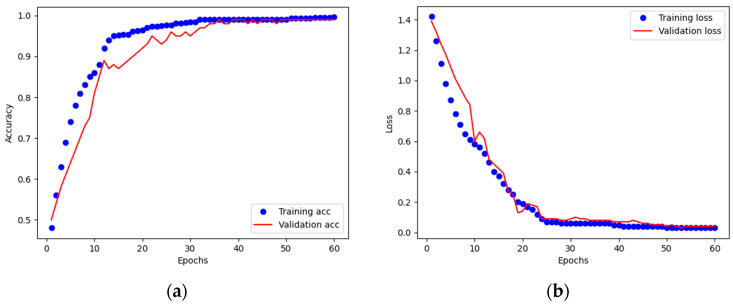
Performance curves: (**a**) Training and validation accuracy; (**b**) Training and validation loss.

Model convergence was carefully monitored through training and validation learning curves shown in Figure 3. The curves demonstrate a smooth increase in accuracy and a consistent decrease in loss across epochs, with no indication of divergence between training and validation performance. The close alignment of the curves reflects stable learning dynamics without evidence of overfitting. Furthermore, an early stopping mechanism based on validation loss was implemented, whereby training would be terminated if the validation loss failed to improve over five consecutive epochs. Notably, this condition was not triggered during the experiments, as the validation loss continued to decrease until the end of training.

These results confirm that the training process achieved robust convergence. The incorporation of augmentation and early stopping, coupled with the absence of loss escalation in the validation set, provides strong evidence that overfitting was effectively controlled despite the limited dataset size.

### 4.2. Detection Performance

The MobileNet V2 Lite model was evaluated for its ability to localize premolar and molar regions on the test set. As summarized in Table 2, the model achieved a Precision of 0.99, Recall of 0.99, and an F1–score of 0.99. These results indicate a high level of agreement between predicted and ground-truth bounding boxes, with both FP and FN detections remaining limited. To further evaluate spatial localization accuracy, the AP was also calculated at fixed IoU thresholds of 0.50 and 0.75. As can be seen in Table 2, The model obtained an AP50 of 0.99 and an AP75 of 0.89. The decrease observed at the stricter IoU threshold reflects the increased sensitivity of this metric to minor spatial deviations in bounding box alignment. Such variations are expected in object detection tasks, particularly when anatomical boundaries are not sharply defined in panoramic radiographs.

The AP75 value indicates that a substantial proportion of predicted bounding boxes maintained strong spatial overlap with the corresponding ground-truth annotations. The high AP50 further confirms that the model consistently identified the relevant tooth regions under standard localization criteria.

The slight discrepancy between AP50 and AP75 can be attributed to marginal boundary shifts in predicted bounding boxes rather than systematic localization errors. Importantly, these minor deviations did not compromise the integrity of the extracted regions of interest. The detector consistently produced tightly bounded ROIs that adequately enclosed the anatomical structures of interest, thereby ensuring that the subsequent classification stage received accurate and standardized inputs.

Overall, the detection results suggest that the model was able to reliably extract regions of interest with satisfactory spatial accuracy. This level of localization performance provides a stable input for the subsequent classification stage without introducing substantial spatial misalignment.

### 4.3. Classification Performance and Overall System Accuracy

Following ROI extraction, the classification network was evaluated on the independent test set for its ability to distinguish between infraocclusion and non-infraocclusion cases. To provide statistically robust performance estimates, 95% confidence intervals (CIs) were calculated using the Wilson score method, which is considered more reliable for proportion-based metrics, particularly in high-accuracy settings [36].

As summarized in Table 3, the overall system achieved an Accuracy of 98.78%, with the CI ranging from 97.55% to 99.42%. The F1–score was also 0.9878, with the same CI range, indicating a balanced trade-off between Precision and Recall. The Recall reached 0.9960, with a CI of 98.60–99.80%, suggesting that the model was able to correctly identify nearly all infraocclusion cases in the test set. Specificity was 0.9800, with a CI of 96.64–99.07%, showing a low rate of FP predictions. The AUC was 0.9986, with the CI ranging from 98.86% to 99.98%, indicating strong discriminatory capability across different classification thresholds. The narrow confidence intervals across all metrics suggest stable performance with limited variability in the test set.

**Figure 4:**
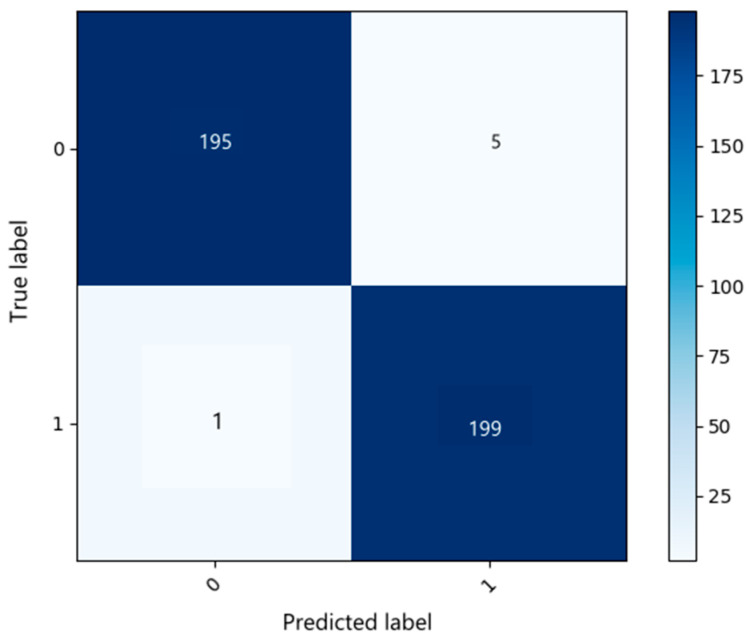
Confusion matrix for the test set.

The classification results suggest that the proposed framework provides reliable discrimination between infraocclusion and no-infraocclusion cases within the evaluated dataset. To further illustrate the predictive behavior of the model, the confusion matrix for the test set is presented in Figure 4. Here, the test set consisted of 400 images, equally distributed between infraocclusion (200) and no infraocclusion (200) cases. As shown in Figure 4, the model correctly classified 195 out of 200 no-infraocclusion cases and 199 out of 200 infraocclusion cases, resulting in a low number of misclassifications across both classes. These findings are consistent with the high Recall and Accuracy values reported in Table 3.

**Figure 5:**
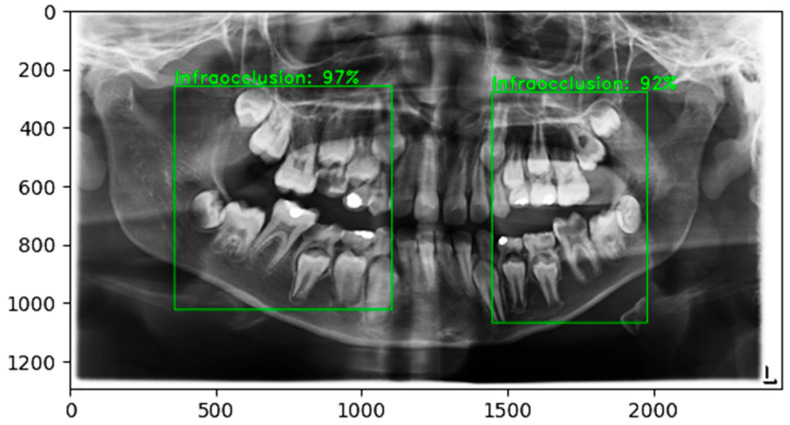
Representative output of the proposed detection and classification framework.

The reliability of the model was further examined through class-wise performance analysis. The classification accuracy was 99.25% for infraocclusion cases and 98.31% for no-infraocclusion cases. The small difference between class-specific accuracies indicates a consistently high level of discrimination across both categories. Given that the test dataset was balanced, this slight variation is likely attributable to natural variability in prediction confidence rather than class distribution bias. Overall, the model demonstrates stable performance across both clinical categories. Figure 5 illustrates the end-to-end output of the proposed framework, where detected regions are subsequently classified as infraocclusion.

### 4.4. Computational Performance

To assess the computational efficiency of the proposed two-stage framework, both the detection and classification modules were evaluated in terms of training time, number of parameters, model size, and inference latency. A summary of these metrics is provided in Table 4.

As can be seen from the table, the classification module, implemented as a custom CNN architecture, contained 1.88 million trainable parameters with a total model size of 7.19 MB. The network required 6 min (min) to complete training. During inference, the average processing time was 0.8 milliseconds (ms) per image, indicating a lightweight structure suitable for rapid deployment.

The detection module, based on SSD MobileNet V2 FPNLite (320 × 320), comprised around 4.48 million (M) parameters with a total model size of 21.82 MB. Training was completed in 39 min, and the inference latency was measured as 19 ms per image. These results demonstrate that the detector maintains computational efficiency despite performing spatial localization in addition to classification.

It can be deduced from the results that both stages exhibit low memory requirements and fast inference latency. The overall computational profile of the proposed framework supports its feasibility for integration into clinical workflows, particularly in environments with limited computational resources.

### 4.5. Comparative Analysis

Successful clinical integration of AI tools in dentistry requires both high diagnostic performance and efficient resource utilization. While accuracy is essential for ensuring reliable diagnosis, computational efficiency directly affects the applicability of such systems in real-world workflows, particularly in pediatric dental settings where timely decision-making is critical. Therefore, a comparative analysis was also conducted to assess the computational feasibility of the proposed framework.

For the detection stage, the performance of the SSD MobileNet V2 FPNLite (320 × 320) used in the proposed framework was compared with Faster R-CNN ResNet50 V1 (640 × 640) [37,38], representing the commonly used configuration in recent two-stage dental imaging studies [26,27]. The comparison results are listed in Table 5. As shown in the table, both models achieved equivalent Precision, Recall, F1–score, and AP50 values (all 0.99), indicating highly accurate object localization at the standard IoU threshold of 0.5 At the stricter AP75 threshold, Faster R-CNN achieved 0.92, while SSD MobileNet V2 obtained 0.89. Although this corresponds to a slight reduction in performance under tighter overlap requirements, the difference may not substantially affect practical ROI isolation for the classification stage.

On the other hand, MobileNet V2 Lite reduced training time from 115 min to 39 min and inference latency from 44 ms to 19 ms. Considering the comparable AP50 performance and near-equivalent AP75 values, this trade-off suggests a more favorable balance between localization accuracy and computational efficiency, particularly for deployment in resource-constrained clinical environments.

For the classification stage, the proposed custom CNN was compared against EfficientNet variants (B0, B3, B6) [34], Inception V3 [39] employed as the backbone for classification in [26], and ResNext50 [40] employed as the backbone for classification in [27]. Table 6 summarizes comparison between the baseline models and proposed CNN in terms of computational performance. As can be seen from the table, the proposed custom CNN model exhibits substantially lower computational complexity compared to all baseline models. The proposed model required around 8 min to complete training, which is considerably faster than ResNext50 (around 25 min), Inception V3 (around 26 min), and EfficientNetB6 (around 44 min). Moreover, with only 1.88 M parameters and a total size of 7.19 MB, it is considerably lighter than ResNext50 (19.73 M parameters, 79.43 MB) and Inception V3 (21.88 M parameters, 83.38 MB), as well as the EfficientNet variants. Furthermore, inference latency was also minimized, reaching 0.8 ms, equal to EfficientNet-B0 and faster than both EfficientNetB3 (1 ms) and EfficientNetB6 (1.3 ms). From a clinical feasibility perspective, the low latency provided by the proposed model may allow predictions to be generated almost instantaneously once the panoramic radiograph is loaded. In practical terms, a dentist could obtain diagnostic feedback within a fraction of a second, supporting real-time or chairside decision-making without disrupting the clinical workflow. This level of responsiveness suggests that the proposed framework could be integrated into existing dental imaging software or clinical information systems.

A comparison between the proposed custom CNN and baseline models in terms of classification performance is compared in Table 7. As shown in the table, the proposed CNN achieved 98.78% overall accuracy, outperforming Inception V3 (88.24%), ResNext50 (87.40%), and EfficientNetB6 (93.25%). Importantly, the proposed model achieved 99.25% accuracy for infraocclusion and 98.31% accuracy for no-infraocclusion cases, whereas EfficientNetB0 completely failed to generalize and EfficientNetB3 achieved imbalanced performance. In terms of error minimization, the proposed CNN also yielded the lowest loss (0.05) compared to other models. Furthermore, the proposed CNN achieved the highest Recall (0.99), F1–score (0.99), and Specificity (0.98). Furthermore, the model reached an AUC of 0.99 that reflects the highest discriminative capability across decision thresholds.

On the other hand, to quantitatively assess the differences in performance between the proposed CNN and baseline models, statistical tests were conducted. DeLong’s test was used to evaluate whether the observed differences in AUC values between correlated ROC curves were statistically significant [41]. In parallel, McNemar’s test was employed to compare paired classification outcomes across models [42]. Additionally, 95% Wilson CIs were computed for all accuracy metrics to account for the precision of estimates. From the results shown in Table 3 and Table 8, it is clear that the proposed CNN achieved a significantly higher AUC and Accuracy compared to Inception V3 and EfficientNet variants (p < 0.05 for both DeLong’s and McNemar’s tests). These results confirm that the performance improvements achieved by the proposed model are statistically significant rather than due to random variation.

The results indicate that while two-stage deep learning frameworks have been previously applied in dental imaging, the proposed framework achieves a more favorable balance between diagnostic performance and computational complexity. The reduced parameter count, lower memory footprint, and faster inference suggest improved feasibility for deployment in resource-constrained clinical environments without compromising predictive performance.

### 4.6. Interpretability

The integration of XAI into medical imaging is increasingly recognized as a prerequisite for clinical adoption, as it enables clinicians to understand, verify, and trust model outputs [43]. Recent works have emphasized that interpretability is essential not only for fostering transparency but also for supporting regulatory approval and for mitigating liability concerns in diagnostic applications [44,45]. Within dentistry, the need for XAI has been explicitly highlighted as a means to bridge the gap between automated decision support and clinical trust, where patient safety and accountability remain paramount [46,47,48,49,50].

**Figure 6:**
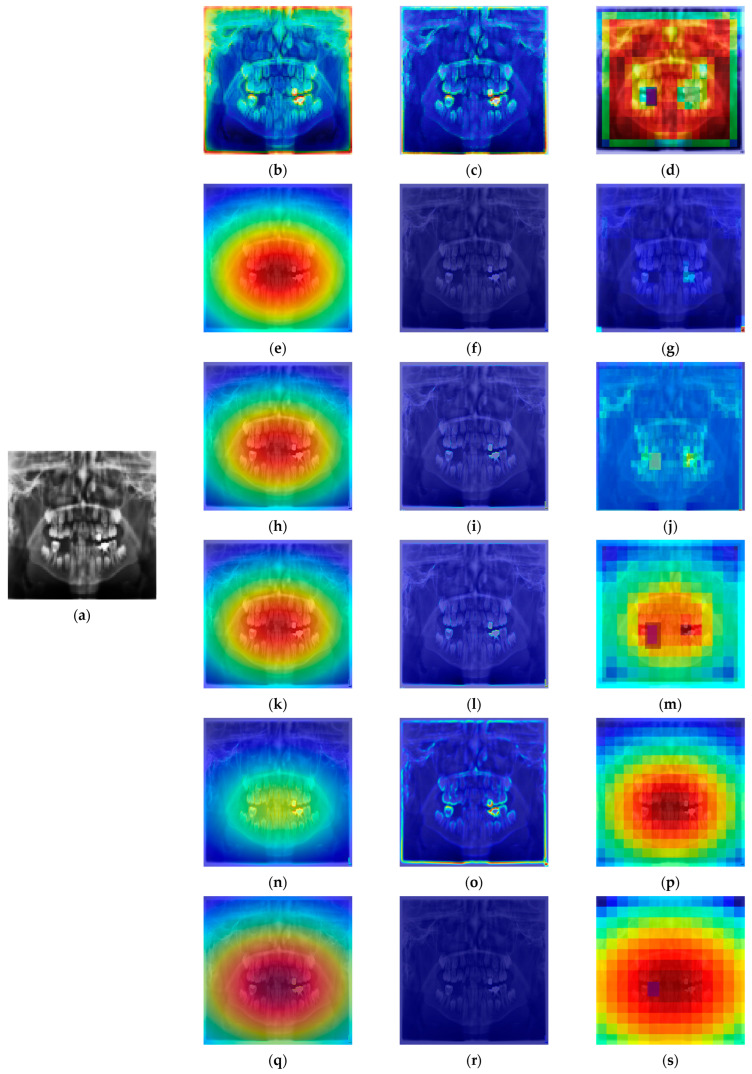
XAI visualizations for an infraocclusion case: (**a**) Original sample; (**b**) Grad-CAM (Custom CNN); (**c**) SmoothGrad (Custom CNN); (**d**) OSM (Custom CNN); (**e**) Grad-CAM (EfficientNetB0); (**f**) SmoothGrad (EfficientNetB0) (**g**) OSM (EfficientNetB0); (**h**) Grad-CAM (EfficientNetB3); (**i**) SmoothGrad (EfficientNetB3); (**j**) OSM (EfficientNetB3); (**k**) Grad-CAM (EfficientNetB6); (**l**) SmoothGrad (EfficientNetB6); (**m**) OSM (EfficientNetB6); (**n**) Grad-CAM (Inception V3); (**o**) SmoothGrad (Inception V3); (**p**) OSM (Inception V3); (**q**) Grad-CAM (ResNext50); (**r**) SmoothGrad (ResNext50); (**s**) OSM (ResNext50).

In this study, Grad-CAM was adopted as the interpretability technique due to its widespread use and effectiveness in localizing discriminative image regions in deep learning models [51]. Comparative analyses have shown Grad-CAM and related methods to be robust for visualizing decision pathways in medical imaging, while also acknowledging their limitations in resolution and faithfulness [52]. To further ensure reliability, we also adopted SmoothGrad [53], which is a saliency-based visualization technique in the field of XAI, and OSM [54], providing a multi-perspective understanding of the decision process. Figure 6 shows the XAI visualizations for an infraocclusion case.

For the proposed CNN, both the Grad-CAM (Figure 6b) and SmoothGrad (Figure 6c) visualizations demonstrate focused attention maps. The highest activations, indicated by the yellow/red regions, are concentrated around the dental arches. These critical regions are particularly located in the vicinity of the occlusal plane and the roots of the erupted and unerupted teeth. This suggests that the proposed model localizes its decision-making to anatomically and clinically relevant regions. Specifically, this region involves the structural alignment and positioning of the teeth within the mandible and maxilla. The OSM (Figure 6d) further corroborates these findings. The regions whose occlusion leads to the greatest drop in confidence, represented by a deep red/yellow overlay, are primarily centered over the central incisors and surrounding alveolar bone. The grid-like structure of the occlusion map highlights a distributed dependency on the specific local features in the lower and upper central dental region. This supports the interpretation that the misalignment or structural anomaly in the occlusal relationship is the key feature being detected by the proposed model.

To further examine the interpretability characteristics across architectures, the XAI analyses were also performed for EfficientNetB0, EfficientNetB3, EfficientNetB6, Inception V3, and ResNext50 according to the XAI visualizations shown in Figure 6e–s. A qualitative comparison of the spatial distribution, localization specificity, and anatomical coherence of activation maps was conducted.

In EfficientNetB0, Grad-CAM visualizations showed broader activation patterns extending across a considerable portion of the dental arch (Figure 6e). Although the infraocclusion region was encompassed within the activated area, it was not distinctly isolated from surrounding dental structures. SmoothGrad maps displayed lower contrast and less sharply defined focal points (Figure 6f). The OSM analysis suggested a more distributed reliance across multiple regions rather than a concentrated dependency on the infra-positioned tooth (Figure 6g). These findings indicate lower localization specificity and higher spatial diffusion compared with the custom CNN model.

EfficientNetB3 demonstrated similar behavior. Grad-CAM activations appeared symmetrically distributed across the central dental region, with limited confinement to the precise pathological site (Figure 6h). The attention patterns were comparatively diffuse (Figure 6i). The OSM indicated that multiple regions along the dental arch contributed to the final prediction, suggesting that the model may have leveraged more global morphological features rather than a sharply localized cue (Figure 6j).

For EfficientNetB6, activation maps remained relatively centralized but covered a broader spatial area than those of the Custom CNN (Figure 6k). SmoothGrad visualizations exhibited patch-like intensity distributions without a clearly dominant hotspot exclusively corresponding to the infraoccluded tooth (Figure 6l). The OSM analysis similarly reflected distributed importance across adjacent anatomical structures (Figure 6m). Localization specificity was moderate, with partial but not exclusive emphasis on the pathological region.

Inception V3 demonstrated moderately localized activation compared to the EfficientNet variants. The infraocclusion site appeared within higher-intensity zones in Grad-CAM maps (Figure 6n). However, attention remained partially distributed across neighboring structures (Figure 6o). The OSM indicated a degree of localized importance, though dependency was not strictly confined to the infraoccluded tooth (Figure 6p). This pattern suggests moderate anatomical coherence and moderate spatial diffusion.

ResNext50 exhibited relatively widespread Grad-CAM activations throughout the dental arch (Figure 6q). The highlighted areas lacked strong spatial confinement, and SmoothGrad maps suggested reliance on broader structural patterns rather than a focal pathological region (Figure 6r). The OSM revealed comparatively limited localized drops in prediction confidence, implying that multiple regions contributed to the classification decision (Figure 6s). Consequently, this architecture demonstrated low localization specificity and higher spatial diffusion.

As a summary, a qualitative comparison of localization specificity, anatomical coherence, and spatial diffusion across models is presented in Table 9. Overall, while all evaluated architectures demonstrated some degree of attention toward clinically relevant dental regions, the proposed custom CNN exhibited comparatively more spatially confined and anatomically coherent activation patterns in this representative infraocclusion case. In contrast, several pretrained backbone models appeared to rely more heavily on globally distributed structural cues rather than on a sharply localized pathological region. These findings suggest that architecture-specific inductive biases may influence the extent to which model decisions align with clinically interpretable anatomical features.

The interpretation of XAI visualizations was qualitatively performed by two experienced pediatric dentists among the authors. In the representative infraocclusion cases analyzed, the highlighted regions were deemed anatomically plausible and consistent with established radiographic diagnostic criteria. The focal activation patterns observed in the proposed model were interpreted as aligning with clinically relevant occlusal discrepancies and infra-positioned tooth structures.

It should be emphasized that XAI techniques such as Grad-CAM, SmoothGrad, and OSM provide approximations of model attention rather than direct representations of causal reasoning. Consequently, variations in the spatial extent, intensity, and boundary sharpness of activation maps may arise due to image-specific characteristics, architectural design differences, and the hierarchical level of feature abstraction within each model. In cases where radiographic findings are subtle or anatomical boundaries are not clearly delineated, interpretation of both the radiograph and the corresponding explanation map may inherently involve a degree of subjectivity.

In addition to the representative example presented, multiple samples from the test set were qualitatively reviewed by the same clinicians. Across these cases, no consistent or systematic discordance between the XAI visualizations and clinical interpretation was observed. Although minor spatial variability was noted across different architectures, the highlighted regions were generally regarded as anatomically plausible within the context of infraocclusion assessment.

## 5. Discussion

### 5.1. Comparison to Prior Study and Clinical Relevance

This study presents a two-stage, clinically oriented deep learning framework specifically adapted for infraocclusion detection in pediatric panoramic radiographs. Rather than introducing a novel convolutional architecture, this study primarily contributes a system-level design consisting of a lightweight, modular pipeline that is optimized for diagnostic reliability, computational efficiency, and practical integration into pediatric dental workflows.

Infraocclusion assessment in pediatric radiographs presents unique challenges, including mixed dentition stages, anatomical overlap, and projection-related distortions that may obscure subtle vertical discrepancies. Early-stage infraocclusion, in particular, may exhibit limited radiographic contrast relative to adjacent structures. The proposed two-stage structure, consisting of ROI localization followed by focused classification, addresses this problem by isolating the tooth-level region prior to diagnostic inference, thereby reducing background noise and improving task-specific feature extraction.

Compared with the only prior study addressing automated infraocclusion detection [26], the proposed framework demonstrates clear performance and efficiency advantages. MobileNet V2 Lite used in the detection module achieved Precision, Recall, F1–score, and AP50 values of 0.99, with an AP75 of 0.89. While Faster R-CNN ResNet50 V1 demonstrated a slightly higher AP75 (0.92), both models produced identical AP50 values (0.99), indicating equivalent detection reliability at clinically relevant IoU thresholds. The marginal AP75 difference reflects tighter bounding-box alignment rather than a meaningful difference in tooth-level localization. Given that infraocclusion diagnosis depends on correct tooth identification rather than pixel-exact contour delineation, the localization performance of the lightweight MobileNet V2 Lite can be considered clinically adequate. Importantly, this comparable localization performance was achieved with substantially lower computational cost. Training time was reduced from 115 min to 39 min, and inference latency from 44 ms to 19 ms. These reductions are particularly relevant for deployment in resource-constrained clinical environments, where GPU availability, memory capacity, and real-time responsiveness may be limited.

In the classification stage, the proposed custom CNN achieved an overall accuracy of 98.78% and an AUC of 0.99, outperforming the classifier employed in [26], Inception V3 (88.24%). This accuracy was obtained with only 1.88 M parameters (7.19 MB), which is substantially smaller than Inception V3 (21.88 M). Training time was reduced to 8 min compared to 26 min for Inception V3, and inference latency decreased to 0.8 ms from 1.3 ms, further supporting near real-time clinical applicability. These findings indicate that high diagnostic performance may not necessarily require high-complexity architectures. Rather, task-adapted lightweight designs can provide a more favorable balance between accuracy and operational feasibility.

### 5.2. Clinical Workflow Integration

A significant advantage of the proposed framework is its modular structure. By decoupling detection and classification, each component can be independently optimized or replaced without disrupting the overall workflow. This design enables future integration of more advanced detection backbones or transformer-based classification models, ensuring scalability and adaptability to evolving AI methodologies.

From a practical standpoint, integration into routine pediatric dental workflows is feasible. Since panoramic radiography is already standard practice, the framework could be incorporated into existing digital imaging pipelines without requiring additional procedures. The system may function as a decision-support layer embedded within radiography software, automatically analyzing images in the background and providing real-time localization and classification outputs prior to clinician review. Rather than replacing clinical judgment, the AI output would serve as an adjunctive alert system, particularly valuable in borderline or equivocal cases where early infraocclusion may be subtle.

To address potential barriers to adoption, such as clinician trust, liability concerns, and institutional variability, XAI techniques were integrated into the framework. By generating localized, human-interpretable visual explanations, the system enhances transparency and clinical plausibility. Nevertheless, further research into multi-method interpretability strategies and user-centered visualization design is essential to strengthen practitioner confidence and facilitate broader acceptance.

### 5.3. Limitations and Future Directions

Despite the promising results, several limitations should be acknowledged. First, mild infraocclusion cases remain diagnostically challenging. Early-stage presentations may exhibit subtle vertical discrepancies that are difficult to distinguish from physiological eruption variations, particularly in panoramic images affected by magnification and projection artifacts. Although overall performance metrics were high, sensitivity in borderline cases may be reduced. Future work should focus on improving early-stage detection through higher-resolution inputs, multi-view imaging strategies, and severity-aware training paradigms. In this context, extending the framework toward a multi-class classification model capable of differentiating mild, moderate, and severe infraocclusion may enhance clinical decision granularity and improve early intervention planning.

On the other hand, the single-center design and the relatively limited sample size may limit the external validity and generalizability of the reported findings. The reported metrics should therefore be interpreted as evidence of methodological feasibility rather than definitive clinical performance. Model behavior may be influenced by institution-specific imaging protocols, device characteristics, and patient demographics. External validation on multi-center datasets encompassing heterogeneous radiographic systems and diverse populations is necessary to evaluate robustness. Prospective cross-institutional studies, combined with domain adaptation techniques, are required to ensure reliable clinical translation and broader applicability.

## 6. Conclusions

This study presents a clinically oriented, two-stage deep learning approach, developed in a controlled experimental setting using a single-center dataset, for the automated detection of infraocclusion in pediatric panoramic radiographs. The two-stage approach, combining MobileNet V2 Lite for the detection with a custom CNN classifier for infraocclusion identification, achieved high diagnostic performance (98.78% overall accuracy), significantly outperforming existing benchmark models.

The results highlight the potential of AI to assist clinicians in the early and reliable identification of infraocclusion, a condition often overlooked in routine practice due to its subtle radiographic presentation. The modular and lightweight architecture enhances practical applicability, facilitating potential integration into chairside diagnostic systems or mobile health platforms. Furthermore, the incorporation of XAI techniques improves interpretability and clinical transparency, which are critical prerequisites for real-world implementation.

As a proof-of-concept study based on a single-center dataset, external validation across diverse clinical environments remains essential prior to deployment. Future research may therefore focus on large-scale, multi-center validation to evaluate robustness and generalizability across diverse radiographic systems, institutional protocols, and patient populations. In parallel, the framework may be extended toward a multi-class classification model capable of distinguishing mild, moderate, and severe infraocclusion. Such refinement, coupled with improved sensitivity for early-stage cases, is expected to enhance granularity in clinical decision support and the translational impact of the proposed framework.

## Figures and Tables

**Table 1:** Subject-level partitioning of the augmented classification dataset across training, validation, and testing subsets.

Dataset Split	Images with Infraocclusion	Images with No Infraocclusion	Total Number of Samples
Training set	933	933	1866
Validation set	201	200	401
Test set	200	200	400
Total	1334	1333	2667

**Table 2:** Detection performance of the MobileNet V2 Lite model.

Metrics	Precision	Recall	F1–score	AP50	AP75
**Value**	0.99	0.99	0.99	0.99	0.89

**Table 3:** Classification performance of the proposed model with 95% Wilson confidence intervals.

Metrics	Estimate	95% Wilson CI (%)
Accuracy	98.78%	[97.55, 99.42]
F1–score	0.9878	[97.55, 99.42]
AUC	0.9986	[98.86, 99.98]
Recall	0.9960	[98.60, 99.80]
Specificity	0.9800	[96.64, 99.07]

**Table 4:** Computational performance of the detection and classification modules.

Stage	Parameters (M)	Model Size (MB)	Training Time (min)	Inference (ms)
Detection	4.48	21.82	39	19
Classification	1.88	7.19	8	0.8

**Table 5:** Comparison between the Faster R-CNN Res-Net50 V1 and SSD MobileNet V2 FPNLite.

Metrics	Faster R-CNN ResNet50 V1	SSD MobileNet V2 FPNLite
Training Time (min)	115	39
Inference Latency (ms)	44	19
Precision	0.99	0.99
Recall	0.99	0.99
F1–score	0.99	0.99
AP50	0.99	0.99
AP75	0.92	0.89

**Table 6:** Comparison between the baseline models and proposed CNN in terms of computational performance.

Metrics	EfficientNetB0	EfficientNetB3	EfficientNetB6	Inception V3	ResNext50	Proposed CNN
Training Time (min)	10	16	44	26	25	8
Total Parameters (M)	4.13	10.89	41.10	21.88	19.73	1.88
Size of Total Parameters (MB)	15.43	41.18	156.29	83.38	79.43	7.19
Inference Latency (ms)	0.8	1	1.3	1.3	1.2	0.8

**Table 7:** Comparison between the baseline models and proposed CNN in terms of classification performance.

Metrics	EfficientNetB0	EfficientNetB3	EfficientNetB6	Inception V3	ResNext50	Proposed CNN
Accuracy (%)	50	61.75	93.25	88.24	87.40	98.78
Loss	0.88	0.71	0.31	0.38	0.47	0.05
Accuracy for Infraocclusion (%)	0	28.00	88.5	86.48	80.80	99.25
Accuracy for No infraocclusion (%)	100	95.50	98	90	94	98.31
F1–score	0.50	0.62	0.93	0.88	0.87	0.99
Specificity	0	0.28	0.88	0.86	0.94	0.98
Recall	1	0.95	0.98	0.90	0.80	0.99
AUC	0.5	0.62	0.96	0.96	0.93	0.99

**Table 8:** Statistical comparison of baseline models against the proposed CNN.

Model	95% Wilson CI		p-Value	
	AUC	Accuracy (%)	DeLong’s Test	McNemar’s Test
EfficientNetB6	[0.9083, 0.9528]	[0.9099, 0.9606]	0.042	0.049
EfficientNetB3	[0.5635, 0.6628]	[0.5736, 0.6598]	0.0009	0.0015
EfficientNetB0	[0.4912, 0.5088]	[0.4991, 0.5009]	0.00002	0.00003
Inception V3	[0.8492, 0.9128]	[0.8507, 0.9087]	0.0019	0.0023
ResNext50	[0.8391, 0.9039]	[0.8378, 0.9025]	0.0021	0.0024

**Table 9:** Cross-architecture qualitative assessment of XAI localization characteristics in an infraocclusion case.

Model	EfficientNetB0	EfficientNetB3	EfficientNetB6	Inception V3	ResNext50	Proposed CNN
Localization Specificity	Low-Moderate	Low	Moderate	Moderate	Low	High
Anatomical Coherence	Moderate	Moderate	Moderate	Moderate	Low-Moderate	High
Spatial Diffusion	High	High	Moderate	Moderate	High	Low

## Data Availability

The data supporting the results of this study may be available from the corresponding author upon request due to privacy and ethical restrictions.
